# Predicting Psychosocial Health of Children and Adolescents with Obesity in Germany: The Underappreciated Role of Physical Fitness

**DOI:** 10.3390/ijerph182111188

**Published:** 2021-10-25

**Authors:** Nina Eisenburger, David Friesen, Fabiola Haas, Marlen Klaudius, Lisa Schmidt, Susanne Vandeven, Christine Joisten

**Affiliations:** Department for Physical Activity in Public Health, Institute of Movement and Neurosciences, German Sport University Cologne, Am Sportpark Müngersdorf 6, 50933 Cologne, Germany; d.friesen@dshs-koeln.de (D.F.); f.haas@dshs-koeln.de (F.H.); m.klaudius@dshs-koeln.de (M.K.); ernaehrungschmidt@gmail.com (L.S.); canollo@web.de (S.V.); c.joisten@dshs-koeln.de (C.J.)

**Keywords:** childhood obesity, health-related quality of life, self-concept, self-perception, physical fitness, psychosocial health

## Abstract

*Background*: The aim of this study was to analyze the inhibitory and promotive factors of psychosocial health in the context of childhood obesity, incorporating physical fitness as an additional, potentially relevant predictor. *Methods*: The sample comprised cross-sectional data of 241 children and adolescents with obesity and overweight from the German Children’s Health InterventionaL TriaL III program (12.5 ± 2.1 years; 51.9% girls). Demographics and lifestyle patterns were assessed via parent reports. Anthropometric data and physical fitness in relation to body weight (W/kg) were measured. Children and adolescents completed standardized questionnaires (GW-LQ-KJ, FSK-K) to assess health-related quality of life (HRQOL) and five dimensions of self-concept (scholastic, social, physical, behavioral, and self-worth). *Results*: Multiple linear regression analysis showed that HRQOL was significantly related to relative physical fitness (W/kg; *β* = 0.216, *p* = 0.011) as were scholastic (*β* = 0.228, *p* = 0.008) and social self-concept (*β* = 0.197, *p* = 0.023). Increasing body mass index (BMI) *Z*-scores, age, physical activity (hours/day), low parental educational levels, and/or migration background were negatively associated with three subdomains of self-concept (physical, behavioral, self-worth; all *p* < 0.05). *Conclusion*: The results emphasize BMI *Z*-scores, age, physical activity, migration background, and parents’ educational level as relevant predictors of psychosocial health in the context of childhood obesity. Additionally, this study adds physical fitness as a key determinant of HRQOL and self-concept. To enable the development of more effective weight management, therapeutic strategies should therefore consider addressing these aspects and improving physical fitness in particular not only for weight loss but also to strengthen psychosocial health.

## 1. Introduction

Childhood and adolescent obesity is a globally recognized public-health concern [1]. In addition to physical comorbidities, such as the increased risk of developing metabolic syndrome, type 2 diabetes, cardiovascular diseases, orthopedic complications, or increased rates of cancer, among others, a growing body of research has documented the psychosocial burden in affected children and adolescents [2]. Psychosocial impairments, such as a poor self-esteem, a negative self-perception or self-concept, can result in a vicious circle of weight gain [3,4,5,6]. Besides this, a negative self-concept has been found to mediate the inverse relationship between high body mass index (BMI) and health-related quality of life (HRQOL) [7], a multidimensional construct aggregating individuals’ physical and psychological health, emotional state, and social functioning [8]. Already in 2014, Buttitta et al. concluded in a review of HRQOL in children and adolescents with obesity, that scientific findings regarding the obesity-related impairment in all dimensions of HRQOL of children and youth were mostly congruent [9].

Consequently, in addition to weight reduction/stagnation and lifestyle counseling, improving mental health, including HRQOL and self-concept, plays a central role in weight management programs [4,10]. To target therapeutic strategies accordingly, the identification of potential inhibitory and promotive obesity-relevant factors for psychosocial health has become a research priority [8,9,10,11]. In this regard, evidence suggests that sex, age, socioeconomic status, and migration background not only affect the prevalence of obesity in children and adolescents but also their HRQOL and self-concept [8,12,13,14]. Further key determinants of both weight status and psychosocial health are lifestyle patterns, such as level of physical inactivity or sedentary behavior, which is characterized by energy expenditure ≤1.5 metabolic equivalents and predominantly involves prolonged sitting and/or laying (e.g., screen-viewing activities, passive transportation) [15,16]. An objective measure of physical activity levels and sedentary behavior is physical fitness [15]. Thus, prior research indicates that it is important to also include physical fitness in the analysis of psychosocial health [17]. Studies with non-overweight children have yielded promising results in terms of psychosocial improvements associated with increased fitness [18,19,20]. Knowing that physical fitness is a mediator in the relationship between childhood and adolescent obesity and self-concept [21,22] and HRQOL [15,23,24], supports the need to further investigate its predictive potential for the psychosocial health of affected children and adolescents. Therefore, the aim of this cross-sectional analysis was to examine determinants of weight-specific HRQOL and subdomains of self-concept in the context of obesity in childhood and adolescence, while considering physical fitness as an additional potentially relevant predictor. Given the vicious cycle between mental health and weight gain, identifying the factors underlying this dynamic may contribute to the development of more effective weight management strategies and recommendations for improved care.

## 2. Materials and Methods

### 2.1. Sample Description

The data for this cross-sectional analysis came from the Children’s Health InterventionaL TriaL (CHILT) III. CHILT III is an 11-month, family-based, multi-component program for obesity prevention and therapy, registered in the German Clinical Trials Register under ID DRKS00026785. It was launched in 2003 at the German Sport University, Cologne. The target groups were children and adolescents aged 8–16 years with obesity or overweight if displaying cardiovascular risk factors, such as arterial hypertension or hyperlipoproteinemia [25].

**Figure 1:**
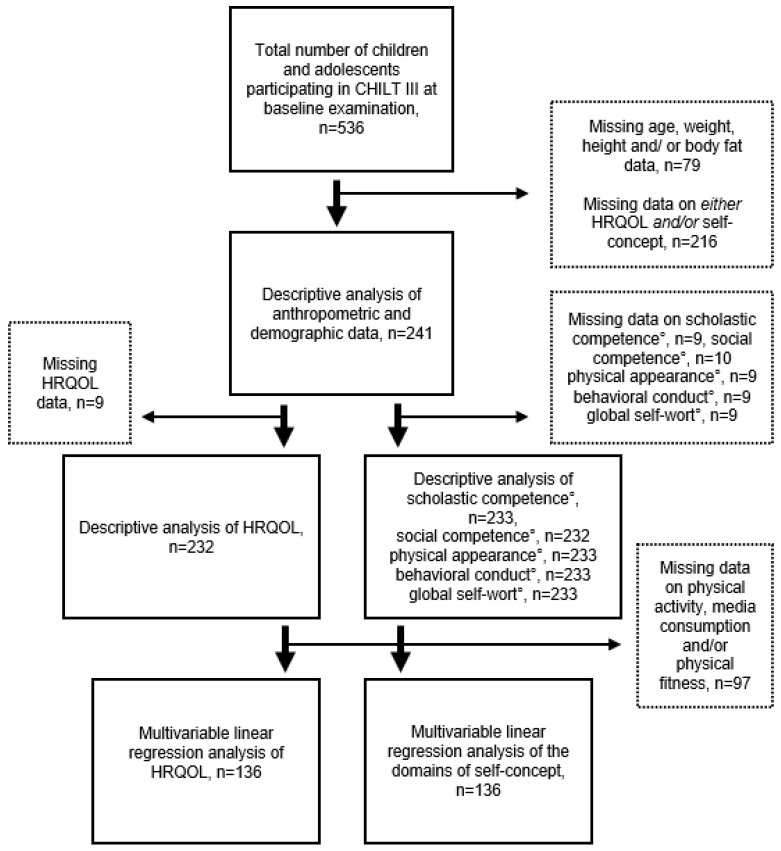
Flow Chart of Number of Participants in the Study; HRQOL, Health-Related Quality of Life; CHILT, Children’s Health InterventionaL Trial; ° subdomains of self-concept; vertical arrows, stepwise procedure of the study; horizontal arrows, stepwise exclusion criteria.

The minimum criteria for inclusion in this study were participation in the CHILT III program between 2003 and 2020; complete height, weight, age, and body fat percentage data at baseline; and fully completed HRQOL and/or self-concept questionnaires. After excluding extreme values, a final data set of 241 children and adolescents (51.9% girls) and their parents (*n* = 459: 236 mothers, 223 fathers) remained (see Figure 1).

### 2.2. Anthropometric Data

Standard calibrated scales and stadiometers were used to measure and weigh each child. Height and weight were measured with the child barefoot. Weight included clothing, such as light sportswear. BMI (weight (kg)/height^2^ (m^2^)) was assessed and sex- and age-specific weight-for-height BMI *Z*-scores were calculated according to the German percentile graphs of Kromeyer-Hauschild et al. [26] using the following equation:

(BMI/M(t))L(t)-1)/(L(t) × S(t)),

where M(t), L(t) and S(t) reflect age- and gender-specific parameters of the child. Children were then categorized as overweight (>90th percentile and ≤97th percentile) or obese (>97th percentile). Body composition was determined by measuring skin-fold thickness to the nearest 0.2 mm in triplicate at the triceps (tric) and subscapula (subs) with a body fat caliper (Harpender Skinfold Caliper HSK-BI, British Indicators, West Sussex, England) following a standardized protocol [27]. The mean of the three measures was considered the final value. When tric+subsc was >35 mm (*n* = 230) the following sex- and age-specific equations by Slaughter (1988) [28] were used to compute the body fat percentage, as these had also been used during previous studies on similar populations [29]:

Girls, %fat = 0.546 × (sum of tric and subs) + 9.7

Boys, %fat = 0.783 × (sum of tric and subs) − 1.7

When tric + subsc was ≤35 mm (*n* = 11), Slaughter’s fat-percentage equations were used according to Rodrígez et al. 2005 [30].

### 2.3. Demographics and Lifestyle Patterns

At the beginning of the program, parents completed standardized questionnaires assessing the demographics and lifestyle patterns of both themselves and their children [25]. Demographic variables selected for inclusion in the study were children’s sex, age, migration background, and parent’s educational background. Time spent in physical activity and on media consumption, which is often used to reflect sedentary behavior, were included to examine lifestyle patterns [31]. Parents’ educational background was dichotomized into two categories: “high”, when both parents had completed secondary school (“Abitur/Fachabitur”), and “low”, when neither parent had an educational degree, a different one other than secondary school, or only one parent had completed secondary school [32]. The migration background of the child was treated as a dichotomous variable assessed by the language spoken at home (German/non-German) [33].

Regarding the child’s level of physical activity, parents were asked if and for how many minutes per week their child was physically active apart from time spent at school. Media consumption was assessed by asking parents to provide the total number of minutes spent by their child per day watching TV, playing a game console, using the computer/Internet, listening to music, and/or using their mobile phone. For this study, media consumption and physical activity were summed and transformed into continuous variables measured in hours per day.

### 2.4. Physical Fitness

Physical fitness was measured in peak mechanical power (PMP (W)) and peak oxygen consumption (VO_2_max (mL/min), data not shown) using a bicycle ergometer (Ergoline Ergometrics 900) on which the children and adolescents exercised until exhaustion. Prior to testing, participants were familiarized with the test procedure and the bicycle ergometer was adjusted individually (height of seat and handlebar position). Testing began with a workload of 25 W and increased by 25 W every 2 min [25]. Throughout the testing session, the participant was verbally encouraged by staff to achieve maximal effort. Due to the comparably larger sample size, peak mechanical power (*n* = 238) was used as a proxy for physical fitness instead of VO_2_max (*n* = 228). Test results were related to body weight as W/kg.

### 2.5. Health-Related Quality of Life

The weight-specific quality-of-life questionnaire for children and adolescents with overweight and obesity (“Fragebogen zur gewichtsbezogenen Lebensqualität für übergewichtige und adipöse Kinder und Jugendliche” (GW-LQ-KJ)) by Warschburger and Fromme (2004) [34] is a self-assessment tool specifically designed to assess the HRQOL of children and adolescents with obesity and overweight. In this study, we used version B of the GW-LQ-KJ which consists of 11 items (e.g., “Because of my weight, I was reluctant to go to the public swimming pool”). The children and adolescents were asked to evaluate the statements by estimating the frequency of occurrence in the last 2 weeks on a five-point Likert scale (ranging from “always” to “never”). The results were recoded so that high values indicated high HRQOL. A summed score was calculated and adjusted to be within a range of 0–100. Dividing the mean individual values by the number of completed questionnaires provided the relative mean. For reliability analysis, Cronbach’s α was calculated. The internal consistency of the HRQOL score of the present sample (*n* = 226) was satisfying, with α = 0.82.

### 2.6. Self-Concept

The FSK-K (“Fragebogen zur Erfassung von Selbst—und Kompetenzeinschätzungen bei Kindern”) is a German version of Harter’s Self-Perception Profile for Children [35] and has been used in previous studies in the context of childhood obesity [36]. It is a 30-item self-report to assess the multidimensional self-concept of children. Each item is scored on a scale of 1–4 in an alternative-statement format, with a positive statement on one side (e.g., “I want to stay the way I am”) and a negative statement on the other side (e.g., “I would like to be someone else”). The child decided which side of the description was “sort of true” or “really true” for him/her.

The FSK-K integrates five scales for assessing perceived domain-specific self-concept: scholastic competence, social competence, physical appearance, behavioral conduct, and global self-worth. After recoding, the highest domain-specific competence was defined as a mean score of 100. Internal consistency of the domains of self-concept was α = 0.79 for scholastic competence (*n* = 231), α = 0.82 for social competence (*n* = 223), α = 0.76 for physical appearance (*n* = 215), α = 0.77 for behavioral conduct (*n* = 228), and α = 0.71 for global self-worth (*n* = 215).

### 2.7. Statistical Analysis

Descriptive statistics for anthropometric data, demographics, lifestyle patterns, and physical fitness are provided. Continuous variables are shown as means ± standard deviation (SD), minimum (min), and maximum (max), and categorical variables as frequencies and percentages. The association between the selected determinants, HRQOL, and the dimensions of self-concept were explored by backward stepwise multiple linear regression analysis, with *p* > 0.05 designating the removal of variables. The dependent variables were the HRQOL score and the score of each of the five domains of self-concept. One model was used for each domain. Predictors included in the baseline model were age, sex, BMI *Z*-score, body fat (%), physical fitness (W/kg), physical activity (hours per day), media consumption (hours per day), migration background (German (yes/no)), and parental educational background (High (yes/no)). A squared term for age was also included as a covariate given that the relationship between HRQOL/(physical) self-concept and age is non-linear [37]. Significance was set at *p* < 0.05. All analyses were performed using IBM SPSS Statistics Version 27.0.

## 3. Results

The average BMI *Z*-score of the sample was 2.45 ± 0.46, with 212 participants classified as obese (88%) and 29 (12%) considered overweight. For a more detailed description of the sample characteristics, see Table 1.

For reference, the six baseline multiple linear regression models explaining HRQOL and the dimensions of self-concept (scholastic competence, social competence, physical appearance, behavioral conduct, and global self-worth) adjusting for all independent variables are shown in Table S1 in the supplementary material. Table 2 summarizes the resulting final models after the removal of all insignificant variables using backward stepwise multiple regression analysis.

After all other factors had been accounted for in the final models explaining HRQOL, scholastic competence, and social competence, relative physical fitness remained the only significant predictor. Participants with high levels of relative physical fitness (W/kg) showed higher HRQOL (*β* = 0.216, *p* = 0.011; Adj. R^2^ = 0.040, *p* = 0.011) and perceived scholastic (*β* = 0.228, *p* = 0.008; Adj. R^2^ = 0.045, *p* = 0.008) and social competence (*β* = 0.197, *p* = 0.023; Adj. R^2^ = 0.031, *p* = 0.023). Relative physical fitness explained approximately 3.1–4.75% of total variability in each of these first three models.

We found BMI *Z*-score and physical activity to be significantly associated with only one of the dependent variables investigated. More precisely, BMI *Z*-score (*β* = −0.334, *p* < 0.001) and self-reported physical activity (*β* = −0.164, *p* = 0.040) significantly predicted physical appearance. Jointly with age (*β* = −0.276, *p* = 0.001), the three predictors accounted for approximately 17% of the total variability in the final physical appearance model (Adj. R^2^ = 0.171, *p* < 0.001).

In the fifth model explaining behavioral conduct, high parental educational levels (*β* = 0.204, *p* = 0.016) and migration background (No/German; *β* = 0.169, *p* = 0.045) showed a positive association to this subdomain of self-concept and explained a significant proportion of variance (Adj. R^2^ = 0.057, *p* = 0.008). Higher parental education was also positively associated with global self-worth (*β* = 0.224, *p* = 0.008), and together with age (*β* = −0.186, *p* = 0.028) accounted for approximately 6% of total variability in the final global self-worth model (Adj. R^2^ = 0.063, *p* = 0.005).

## 4. Discussion

Childhood and adolescent obesity impacts various dimensions of psychosocial health, including health-related quality of life (HRQOL) and personal self-concept [3]. Thus, a comprehensive understanding of the dynamics and possible influencing factors between weight and mental health is a key step toward improving weight management programs. In this context, our results confirmed previous findings on the negative association between increasing age, high BMI *Z*-scores, migration background, low parental education, and psychosocial health [12,13,32]. In addition, the results revealed that relative physical fitness was a major predictor of HRQOL, and of the social and scholastic self-concept of children and adolescents with overweight and obesity.

### 4.1. Anthropometric and Demographic Determinants of Psychosocial Health of Children and Adolescents with Obesity

Consistently with the literature, we identified the physical self-concept to be most affected by high BMI *Z*-scores [9]. The higher the *Z*-score, the more dissatisfied participants were with their physical appearance in our sample. Many studies have concluded that girls with overweight or obesity are especially susceptible to body dissatisfaction [5]; however, other studies have not yielded results containing differences between the sexes [2]. While we did not find differences related to sex between any dimension of self-concept or HRQOL, we did find age to be a significant predictor in our study. These results are not surprising when considered in the context of the effects of puberty. Pubescent individuals are particularly vulnerable to low self-esteem and negative body image [5,37]. Our findings thus support those of earlier research, which suggested that addressing body image should be included as a highly relevant issue in obesity-treatment agendas to improve patients’ self-esteem, particularly in adolescence [10].

Migration background and low socioeconomic status have been identified as key determinants of obesity and are also associated with determinants of psychosocial health [12,32]. Several researchers have recommended interventions at an early stage in childhood to address children who—due to their familial background—are particularly at risk of developing obesity and psychosocial impairments [4]. In line with this, our findings indicate that children and adolescents with obesity and overweight who have a migration background or whose parents had comparatively low education assessed their behavioral conduct and global self-worth as worse than their German counterparts. Considering the observed age effect in our study, the need for early action becomes especially evident. Besides this, the strong influence of familial background and behavior-specific family variables (e.g., lifestyle patterns and nutrition) in the context of both obesity and the subdomains of self-concept underpin the need for parental involvement in intervention strategies [13].

### 4.2. Associations between Physical Fitness, Physical Activity, and Psychosocial Health of Children and Adolescents with Obesity

In weight management programs, most participating families focus on weight loss as the key determinant of program success [24]. However, when considering the underlying causal relationships between weight gain, active lifestyle, and psychosocial health, the latter should be regarded as equally important outcome measures [9,34,35]. In this regard, our results demonstrate that physical fitness may be an important contributor to achieving program goals beyond mere weight loss. While its positive effect on physical health and weight management is undisputed, this study, on the one hand, identifies the importance of physical fitness for the personal self-concept and, on the other hand, reemphasizes the relevance of physical fitness for HRQOL in childhood [20], adolescence [19,24], and in the context of obesity [23]. Because it is associated with both physical and psychosocial dimensions [18,21,22], our results hence suggest that a focus on improving fitness could lead to more sustainable therapy outcomes than short-term weight loss [17].

It is important to note that objectively measured fitness played a greater role for the selected markers of psychosocial health than subjectively measured physical activity or self-reported media consumption in our sample. In comparison to physical fitness, self-reported media consumption was not a significant predictor in the present analysis. Physical activity was negatively associated solely with perceived physical appearance. The observed negative relationship between physical activity and appearance was not consistent with previous studies [18,22] which may be explained by the fact that engaging in physical activity may reveal fundamental movement-skill difficulties compared to non-overweight peers, leading to an impairment of physical self-concept [14]. Therefore, our results indicate that—in addition to physical fitness improvements—motor skill development in children and adolescents with overweight and obesity may be critical in intervention strategies [14].

### 4.3. Strengths and Limitations

A major strength of our study is that physical fitness, body height, weight, and fat percentage were objectively measured by trained staff according to standardized methods. In addition to the large sample and the number of determinants analyzed, further strength is the utilization of a weight-specific HRQOL-measurement tool that has been shown to have good psychometric properties.

The primary limitation of our study is the cross-sectional design, which does not allow any conclusions to be drawn regarding the causal direction of the relationship between the observed variables. Furthermore, several obesity-relevant factors were not included due to incomplete data such as dietary habits, type of school, single parenting, and parents’ BMI. The inclusion of these limited data would have resulted in too great a sample size restriction. As such, there may be additional factors that could confound the association between the independent variables. Selection bias, information bias, and social desirability bias, that is, in self-reports on physical activity and media consumption, are further limitations. As a treatment-seeking population, the participants potentially shared characteristics, such as motivation, that distinguished them from other groups. Besides this, as some data were self-reported, the study is not free from information bias.

## 5. Conclusions

This study adds to the existing body of research by identifying inhibitory and promotive factors for HRQOL and self-concept in the context of childhood obesity, with implications for therapy and care. The results identify physical fitness as a key predictor of weight-specific HRQOL and subdomains of self-concept of children and adolescents with obesity. These findings suggest that improvements in physical fitness may hold even more promise for positive psychosocial health outcomes in weight management programs than weight loss or participation in physical activity alone. In addition, addressing at-risk children from lower socioeconomic or migration backgrounds at an early stage might be crucial not only to prevent obesity but also to improve mental health. Our findings furthermore indicate that strategies to promote body satisfaction and motor abilities could be critical especially in adolescence to improve the physical self-concept of adolescents with overweight and obesity but future longitudinal studies are required to investigate the robustness and causality of our findings.

## Figures and Tables

**Table 1:** Descriptive Sample Characteristics.

Variable		*n (%)*	Mean (SD)	Min	Max
Sex	Female	125 (51.9%)			
	Male	116 (48.1%)			
Percentile	Obese	212 (88.0%)			
	Overweight	29 (12.0%)			
Migration Background	Yes/German	209 (86.7%)			
	No/Non-German	32 (13.3%)			
Parent’s Educational Degree ^1^	High	57 (23.7%)			
	Low	184 (76.3%)			
Physical Variables	Age (years)	241	12.5 (2.07)	7.3	17.1
	Height (m)	241	1.58 (0.11)	1.23	1.89
	Weight (kg)	241	76.7 (19.9)	37.4	148.4
	BMI (kg/m^2^)	241	30.9 (4.8)	20.5	56.6
	BMI *Z*-score	241	2.45 (0.46)	1.43	3.80
	Body fat (%)	241	42.1 (9.0)	26.1	83.2
	Relative Physical fitness (W/kg)	241	1.7 (0.4)	0.9	3.3
Lifestyle Variables	Physical Activity (hours/day)	162	0.7 (0.6)	0	2.9
	Media Consumption (hours/day)	190	2.5 (1.8)	0	8.5
Psychosocial Variables	HRQOL	232	77.7 (14.3)	29.1	100.0
	Scholastic Competence °	233	75.6 (16.7)	25.0	100.0
	Social Competence °	232	76.0 (18.6)	25.0	100.0
	Physical Appearance °	233	54.1 (15.6)	25.0	100.0
	Behavioral Conduct °	232	74.1 (16.4)	29.2	100.0
	Global Self-Worth °	233	72.3 (16.3)	25.0	100.0

^1^ High, both parents have completed secondary school; Low, only one parent/neither mother nor father have completed secondary school; HRQOL, Health-Related Quality of Life; *n*, number of participants; SD, Standard Deviation; Min, Minimum; Max, Maximum; ° Subdomains of Self-Concept; Psychosocial variables are based on scores ranging from 0 (lowest) to 100 (highest).

**Table 2:** Final Models from Backard Stepwise Multivariable Linear Regression Analysis.

Model	Final Predictor/s *	*β* (s.e.)	*p*-Value of Coefficient	R^2^	Adj. R^2^ (*p*-Value of Final Model)
HRQOL (*n* = 136)	Relative Physical fitness (W/kg)	0.216 (3.128)	0.011	0.047	0.040 (0.011)
Scholastic Competence ° (*n* = 136)	Relative Physical fitness (W/kg)	0.228 (3.289)	0.008	0.052	0.045 (0.008)
Social Competence ° (*n* = 136)	Relative Physical fitness (W/kg)	0.197 (3.786)	0.023	0.038	0.031 (0.023)
Physical Appearance ° (*n* = 136)	Age (years) BMI *Z*-score Physical Activity (hours/week)	−0.276 (0.641) −0.334 (2.726) −0.164 (0.301)	0.001 <0.001 0.040	0.190	0.171 (<0.001)
Behavioral Conduct ° (*n* = 136)	High Parental Educational Level ^a^ German/No Migration Background ^b^	0.204 (2.942) 0.169 (4.442)	0.016 0.045	0.071	0.057 (0.008)
Global Self-Worth ° (*n* = 136)	Age (years) High Parental Educational Level ^a^	−0.186 (0.719) 0.224 (3.005)	0.028 0.008	0.077	0.063 (0.005)

* After removal of all insignificant variables. Significance was set at *p* < 0.05; HRQOL, Health-Related Quality of Life; *β*, Standardized Coeffecient Beta; s.e., Standard Error; Adj, Adjusted; ° Subdomain of Self-Concept; Reference Categories: ^a^ low parental educational level (only one parent/neither mother nor father have completed secondary school/*Abitur*), ^b^ Non-German.

## Data Availability

The data used and analyzed during the current study involve sensitive patient information and indirect identifiers. As a result, the datasets are available from the corresponding author only on reasonable request.

## Supplementary Materials

The following materials are available online at https://www.mdpi.com/article/10.3390/ijerph182111188/s1, Table S1: Baseline Multivariable Linear Regression Models.
