# Supplementary material for: Predicting Psychosocial Health of Children and Adolescents with Obesity in Germany: The Underappreciated Role of Physical Fitness
Source: Int J Environ Res Public Health. 2021 Oct 25;18(21):11188. doi: 10.3390/ijerph182111188 (PMC8583523; doi:10.3390/ijerph182111188)
Supplement: Supplementary file 1 [file ijerph-18-11188-s001.zip › ijerph-1403787-supplementary.pdf]

Table S1. Baseline Multivariable Linear Regression Models

**Variables**		**HRQOL** *n*=136	**Scholastic Competence°** *n*=136	**Social Competence°** *n*=136	**Physical Appearance°** *n*=136	**Behavioral Conduct°** *n*=136	**Global Self-Worth°** *n*=136
Age (years)	β (s.e.)	-0.509 (6.458)	-0.628 (7.192)	-0.539 (8.267)	-1.015 (6.834)	-0.733 (0.7508)	-1.465 (7.705)
	p-value	0.562	0.485	0.551	0.223	0.412	0.104
Age squared (years)	β (s.e.)	0.529 (0.264)	0.482 (0.291)	0.507 (0.335)	0.749 (0.277)	0.612 (0.304)	1.282 (0.312)
	p-value	0.546	0.590	0.574	0.327	0.492	0.153
Female [a]	β (s.e.)	-0.149 (2.533)	-0.090 (2.789)	0.087 (3.205)	-0.083 (2.650)	-0.049 (2.911)	-0.024 (2.987)
	p-value	0.092	0.321	0.341	0.327	0.663	0.787
BMI Z-score	β (s.e.)	-0.077 (3.585)	-0.017 (3.884)	0.214 (4.465)	**-0.290 (3.691)**	0.135 (4.055)	-0.128 (4.161)
	p-value	0.495	0.880	0.064	**0.008**	0.234	0.263
Body fat (%)	β (s.e.)	-0.159 (0.159)	-0.003 (0.176)	-0.179 (0.202)	-0.096 (0.167)	-0.122 (0.184)	-0.027 (0.188)
	p-value	0.164	0.978	0.093	0.332	0.244	0.795
High Parental Educational Level[b]	β (s.e.)	0.117 (2.906)	**0.193 (3.098)**	0.083 (3.561)	0.001 (2.944)	**0.194 (3.234)**	**0.192 (3.319)**
	p-value	0.206	**0.039**	0.371	0.992	**0.037**	**0.040**
German/No Migration Background[c]	β (s.e.)	-0.067 (4.054)	-0.062 (4.373)	-0.043 (5.027)	-0.064 (4.155)	0.157 (4.565)	0.019 (4.685)
	p-value	0.436	0.475	0.620	0.431	0.070	0.828
Relative Physical fitness (W/kg)	β (s.e.)	0.128 (3.750)	0.199 (4.012)	0.197 (4.612)	-0.041 (3.812)	0.134 (4.189)	0.034 (4.298)
	p-value	0.209	0.055	0.059	0.671	0.193	0.742
Physical Activity (hours/day)	β (s.e.)	-0.086 (0.318)	-0.058 (0.341)	0.065 (0.392)	**-0.182 (0.324)**	-0.040 (0.356)	-0.048 (0.365)
	p-value	0.334	0.526	0.479	**0.035**	0.663	0.601
Media Consumption (hours/day)	β (s.e.)	-0.113 (0.103)	0.011 (0.109)	-0.127 (0.126)	-0.122 (0.104)	-0.097 (0.114)	-0.022 (0.117)
	p-value	0.220	0.910	0.177	0.164	0.297	0.811
R^2		**0.134**	0.114	0.103	**0.218**	0.122	0.114
Adj. R^2		**0.065**	0.043	0.031	**0.156**	0.052	0.043
Final Model *p*-value		**0.045**	0.113	0.175	**<0.001**	0.078	0.111

HRQOL, Health-Related Quality of Life; β, Standardized Coefficient Beta; s.e., Standard Error; Adj, Adjusted; °Subdomains of self-concept; Reference Categories: [a] Male, [b] low parental educational level (only one parent/neither mother nor father have completed secondary school/*Abitur*), [c] Non-German; Significance set at $p<0.05$; Significant values in bold
